# Strategies for enacting health policy codesign: a scoping review and direction for research

**DOI:** 10.1186/s13012-023-01295-y

**Published:** 2023-09-21

**Authors:** Sarah Cusworth Walker, Barbara Baquero, Betty Bekemeier, McKenna Parnes, Kashika Arora

**Affiliations:** 1 Department of Psychiatry and Behavioral Sciences, School of Medicine, University of Washington, Box 356560, Seattle, USA; 2 School of Public Health, University of Washington, 3980 15th Ave, Box 351621, Seattle, NE USA; 3 School of Nursing, University of Washington, Box 357263, Seattle, USA; 4 Seattle Children’s Hospital, 6901 Sand Point Way NE, Seattle, WA 98115 USA

**Keywords:** Policy, Codesign, Scoping review, Health, Coproduction

## Abstract

**Background:**

Strategies for supporting evidence-informed health policy are a recognized but understudied area of policy dissemination and implementation science. Codesign describes a set of strategies potentially well suited to address the complexity presented by policy formation and implementation. We examine the health policy literature describing the use of codesign in initiatives intended to combine diverse sources of knowledge and evidence in policymaking.

**Methods:**

The search included PubMed, MEDLINE, PsychInfo, CINAHL, Web of Science, and Google Scholar in November 2022 and included papers published between 1996 and 2022. Terms included codesign, health, policy, and system terminology. Title and abstracts were reviewed in duplicate and included if efforts informed policy or system-level decision-making. Extracted data followed scoping review guidelines for location, evaluation method, health focus, codesign definition, description, level of health system user input, sectors involved, and reported benefits and challenges.

**Results:**

From 550 titles, 23 citations describing 32 policy codesign studies were included from multiple continents (Australia/New Zealand, 32%; UK/Europe, 32%; South America, 14%; Africa, 9%; USA/Canada 23%). Document type was primarily case study (77%). The area of health focus was widely distributed. Policy type was more commonly little p policy (47%), followed by big p policy (25%), and service innovations that included policy-enabled funding (25%). Models and frameworks originated from formal design (e.g., human-centered or participatory design (44%), political science (38%), or health service research (16%). Reported outcomes included community mobilization (50%), policy feasibility (41%), improved multisector alignment (31%), and introduction of novel ideas and critical thinking (47%). Studies engaging policy users in full decision-making roles self-reported higher levels of community mobilization and community needs than other types of engagement.

**Discussion:**

Policy codesign is theoretically promising and is gaining interest among diverse health sectors for addressing the complexity of policy formation and implementation. The maturity of the science is just emerging. We observed trends in the association of codesign strategies and outcomes that suggests a research agenda in this area could provide practical insights for tailoring policy codesign to respond to local contextual factors including values, needs, and resources.

Contributions to the literature

- Research shows gaps in the types of evidence preferred by decision-makers and researchers in policy formation processes.
- Codesign is a strategy used to integrate diverse forms of knowledge in health policy formation that has the potential to resolve these challenges.
- The study of codesign within policy is in an early stage, but existing implementation suggests wide appeal globally and across health sectors.
- Results suggest evaluating the relationship between the level of policy user engagement and policy formation outcomes, including community mobilization and knowledge of community needs, will be a productive area of further research.

## Introduction/background

In implementation science, health policies are often conceptualized as outer setting factors that enable improved population health by directing funding towards the implementation of evidence-based interventions (EBIs) [1–3]. The emerging research focused on *policy development strategies* [4, 5], however, suggests “evidence-based” is a less helpful construct than “evidence-informed” [6]. In this policy development subfocus area of implementation science, scholars increasingly recognize the need for pull rather than push strategies to ethically and effectively integrate research evidence from health science into the complex realities of real-world policymaking [7, 8].

This *evidence-informed* perspective also aligns with political science models that explain policy and public administration failures. In one such model, New Public Governance [9, 10], failures are attributed to misalignment among the sectors needed for successful policy implementation (e.g., policymaker, service delivery sector, consumer, community member). The relationship between poor sector alignment and poor EBI implementation is routinely documented in implementation science as well [11]. Updated implementation science frameworks [12, 13] identify the need for collaborative planning rather than top-down strategies when innovations or contexts present high complexity.

Policymaking is one such highly complex decision-making environment. Policymakers and those facilitating policy development have to navigate conflicting constituent values, short time frames, and difficulties accessing timely, relevant knowledge. Many of the questions addressed by policy have multiple causal factors and competing courses of possible action [14]. When surveyed, policymakers note the difficulty of accessing and appropriately applying research evidence given these constraints [15, 16]. As a result, research evidence tends to either be unused or applied in non-optimal ways, posing barriers to implementation successes and health equity.

Codesign is a framework for collaboration potentially well suited to addressing the complexity of multi-sector policymaking. The purpose of codesign is to develop a space for sense-making among individuals with different cultures, beliefs, and forms of knowledge [17]. As articulated by thought-leaders in this field, effective bridging among sectors requires a mindset shift among policy developers from expert to facilitator. Operationally [18], this moves the developer from being the one to gather knowledge and produce recommendations and policies, to one who convenes and creates opportunities for individuals from diverse professional and lived experience backgrounds to create shared understanding and agreement on policy direction. As noted by Evans and Terry [19], common features of design-based models include iterative stages of divergence and convergence, with a series of phases starting with discovery or inspiration, leading to design or ideation, and followed by implementation.

The integrative approach fundamental to codesign provides a model for bringing research evidence together with system knowledge and service user knowledge in an “additive” approach to policymaking [20]. The use of research evidence in this collaborative framework reflects *conceptual* use as articulated by Carol Weiss [21]. Sometimes referred to as enlightenment use, conceptual use describes how research influences the way policymakers and those in the policy arena think about a topic. This may result in a shift of mindset or mental model or may confirm the participants’ view of the problem in a way that enhances motivation to act [22, 23]. This also aligns with the predictions of New Public Governance (NPG) which posits that focusing on the process of policy formation rather than a specific policy structure (e.g., funding an EBP) is expected to produce better downstream outcomes [24, 25]. In NPG, successful policy formation can be evaluated by assessing the extent to which considered policies are scrutinized by multiple perspectives, encompass clearly defined goals and strategies, are descriptively innovative, articulate how the policy could navigate the trade-offs of complex policy problems (wicked problems), are supported by diverse policy stakeholders, and are sufficiently flexible to be adjusted over time [9].

Codesign and similar cocreation approaches are rapidly growing methodologies in health services [26], but the literature on the application of codesign within health policy is limited. Consequently, the claimed benefits of this approach to health-related policymaking are largely theoretical. The complex nature of policymaking suggests the application of any methodology in this area, especially one with the ambitious claims of codesign, requires thoughtful theoretical and empirical scrutiny. The dramatic rise in visibility and popularity of codesign has led to widespread adoption of the term. In a survey of public service workers (*n* = 466), 90% self-reported the use of codesign [27] but proponents of formally described codesign approaches argue that it is rare to find the skills and mindsets for codesign within the public sector [28]. Politically, some are concerned that the use of term codesign in policy spaces is being coopted to provide participants with a false sense of ownership while policy decisions continue to be dictated by more powerful actors [29, 30].

To advance our understanding of codesign as a policymaking strategy in health policy research, we undertook a scoping review of the health policy codesign literature. Our aim was to characterize the existing state of research in this area, analyze available approaches against the claims of guiding theory, and propose recommendations for developing and evaluating codesign as a strategy for policy formation within policy dissemination and implementation science. In doing so, we adopted a broad definition of policy, including Big p, little p, and policy-enabled service development [31]. Big p policies are laws that regulate resources. Little p policies are institutional norms and regulations. Policy-enabled service development includes policymakers or policy levers in service innovation.

## Method

### Purpose

We conducted this scoping review to capture policy codesign strategies across health-related disciplines to examine disciplinary and theoretical origins, activities, and the relationships among strategies and outcomes as reported in existing studies.

### Study design

Scoping reviews are a methodologically rigorous approach to describing scholarly literature on a topic of interest [32]. We used the most recent guidance for conducting high-quality scoping reviews, drawing from foundational literature [32] and updated methods [33–35]. The review was conducted in accordance with the Joanna Briggs Institute methodology for scoping reviews [36]. Article selection and synthesis were conducted based on the Preferred Reporting Items for Systematic Reviews and Meta-Analyses extension for Scoping Reviews (PRISMA-ScR) checklist.

### Information sources and search strategy

We systematically searched relevant published research articles in April 2021 and updated our search in November 2022. Our search involved the following databases: PubMed, Academic Search Complete, Web of Science, EBSCO (MEDLINE, PsychInfo, and CINAHL), and Google Scholar. We stopped reviewing returns from Google Scholar after the first 100 articles. Google sorts by relevance and after the first 30 articles, we did not identify any additional eligible documents. We included text words contained in the titles, abstracts, and keywords of articles. All keywords and index terms were adapted for each database and/or information source and consisted of three levels of included terms. The first level aimed to capture efforts that aspired to use cocreation methodologies using the terms “codesign, cocreation, participatory design, coproduction, and community-based participatory research.” The second level aimed to limit the search to health-related areas with the term “health.” The third level aimed to limit the search to papers regarding policy and complex systems with the terms “complex health, complex public health, policy, community, healthcare system, and system.” The reference section of articles selected for full-text review were also searched, and potentially relevant articles were added to the full-text review list as well.

### Inclusion/exclusion

Included articles described an effort by non-citizen led entities (e.g., government, academia, nonprofit, advocacy) to initiate a process to improve a health-related need through policy or system-level processes. The articles needed to include sufficient information to code the steps and strategies used in the process. Articles had to describe a process that clearly focused on engaging system and policy level changes. Articles not focused on a health or public health need were excluded. We used a broad definition of health, including social determinants of health.

### Procedures

Citations for all identified articles were entered in a Microsoft Excel data worksheet developed by the team for managing systematic reviews. Article titles were reviewed by two independent reviewers for inclusion or exclusion, based on their titles/abstracts until reviewers reached agreement. The remaining title/abstracts were reviewed separately.

### Study selection process

All titles and abstracts were screened independently by pairs of reviewers (SW, KA, BQ) and discrepancies were resolved by the pair. For screening full text articles, one pilot was conducted with 20 articles in which each article was independently reviewed by a pair of reviewers (SW, KA, BQ, BB, MP). When the full group of reviewers reached agreement on screening criteria, full-text articles were independently reviewed by three reviewers (SW, KA, MP) and spot checked by an experienced reviewer (SW).

### Data abstraction

Following Joana Briggs guidelines, we abstracted data on article characteristics (region, type of article) as well as categories for health focus, policy focus, codesign definition, referenced theory, engagement level of policy beneficiaries, multisector involvement, codesign structure (phase, description), perceived benefits, and perceived challenges. Coding within category for health focus and policy focus was developed inductively by each coder. Codes were then recoded into a smaller set of higher-level codes by a single author (SW) and approved by three reviewers (BQ, BB, MP). Coding for codesign definition, theory, engagement of policy beneficiaries, multisector involvement, codesign structure, perceived benefits, and perceived challenges was developed by the process described below.

Data abstraction was conducted using an Excel form developed a priori and pilot-tested on a sample of five papers after which codes were refined. Four reviewers then reviewed a single study (SW, BQ BB, MP), and discrepancies were resolved by consensus. Data were abstracted by one reviewer (KA, MP, SW) and verified by an experienced reviewer for consistency and accuracy (SW).

### Risk of bias assessment

We did not conduct a risk of bias assessment, consistent with Joanna Briggs Institute Scoping Review Methods Manual and scoping reviews on health topics.

### Synthesis of results

Results were synthesized using frequencies and thematic analysis. Thematic analysis was performed by one reviewer (SW) and verified by a second reviewer (BB). Synthesis drew from public policy health concepts [37], community sector literature, citizen participation frameworks [38], and open-coding methods [39]. Consistent with the purpose of scoping reviews, our intent in conducting this study was to characterize the state of the science in the area of health policy codesign.

## Results

### Literature search

**Fig. 1:**
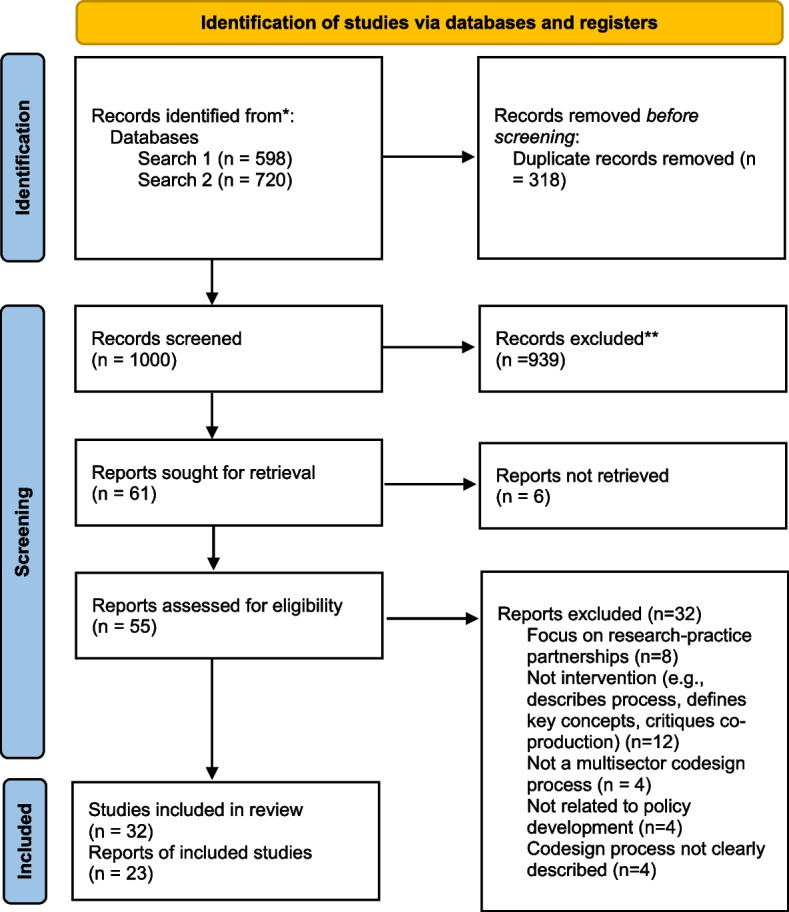
PRISMA diagram

After screening titles and abstracts (550) and full-text documents [40], 23 documents summarizing 32 policy codesign studies met the eligibility requirements (Fig. 1, Table 1). Documents were excluded for describing research-practice partners rather than a time-limited codesign effort (*n* = 32), not providing enough information on approach (*n* = 12), not including multiple sectors (*n* = 4), not relating to policy (*n* = 4), and not describing codesign in sufficient detail (*n* = 4).

**Table 1:** Study characteristics and policy focus

First author last name	Case study	Location	Health area	Health focus	Policy focus
Banana (2015) [41]	Case 1	Zimbabwe	Sanitation	Urban sanitation improvement	New service
Evans (2016) [19]	Case 1	Australia	Family support services	Improve case management for marginalized families	Little p policy
Eppel and Evans (2018) [42]	Case 1	New Zealand	Built environment	Built environment development	Little p policy
Eppel and Evans (2018) [42]	Case 2	New Zealand	Employment	Strategic direction for the whole employment system	Little p policy
Eppel and Evans (2018) [42]	Case 3	New Zealand	Community engagement policy	Strategic direction for community engagement	Little p policy
Eppel and Evans (2018) [42]	Case 4	New Zealand	Social services	Improve social services experience for Maori community	Little p policy
Eppel and Evans (2018) [42]	Case 5	New Zealand	Housing	Prevent rheumatic fever	Little p policy
Eppel and Evans (2018) [42]	Case 6	New Zealand	Financial resiliency	More directly support people experiencing financial hardship	New service
Eppel and Evans (2018) [42]	Case 7	New Zealand	Early childhood education	Increase participation in early childhood education	New service
Holmes (2011) [43]	Case 1	New Zealand	Community economic development	Identify barriers to community economic development	Little and big p policy
Holmes (2011) [43]	Case 5	Australia	Education	Strategic direction in education	Little p policy
Kimbell (2015) [44]	Case 1	UK	Chronic illness	More appropriate services for chronic illness	Big p policy
Llano-Arias, V. (2015) [45]	Case 1	Colombia	Water supply	Strategic direction for maintaining water supply	Big p policy
Llano-Arias, V. (2015) [45]	Case 2	Colombia	Water supply	Water governance	Big p policy
Mullins (2021) [46]	Case 1	Australia	Homelessness	Support for persons without homes	New service
Muñoz-Erickson (2014) [47]	Case 1	Puerto Rico	Urban planning	Urban planning	n/a
Van der Bijl-Brouwe (2016) [40]	Case 1	Australia	Mental health	Support for acute mental illness	Little p policy
Bovaird (2012) [48]	Case 2	UK	Care navigation	Redesigning a social care website	New service
Bovaird (2012) [48]	Case 3	UK	Participatory budgeting	Participatory budgeting for local services	New service
Bovaird (2007) [49]	Case 2	UK	Early child health	Improve readiness for elementary school	New service
Ostrom (1996) [50]	Case 1	Brazil	Urban infrastructure	Construct neighborhood sanitation system	New service
Marchal (2021) [51]	Case 1	Georgia (country)	Tuberculosis policy	Increase tuberculosis treatment	Big p policy
Springs (2019) [52]	Case 1	Rhode Island (US state)	Health services	Identify policies to support arts-based health services	Little p policy
Spaa (2022) [53]	Case 1	UK	Multiple	Multiple	Big p policy
Richardson (2021) [54]	Case 1	UK	Housing	Approach for participatory decision making within a housing development	Little p policy
Young (2018) [55]	Case 1	Canada	Prescription coverage	Payment policy for high-cost drugs for rare disorders	Big p policy
Bittle (2022) [56]	Case 1	USA (county)	Public health	Approach to participatory decision-making in funding	Little p policy
Lloyd-Williams (2021) [57]	Case 1	UK	Non-communicable diseases	Policies needed to prevent NCDs	Big p policy
Goodyear (2022) [58]	Case 1	Austria	Mental health	Policy and practice related to child mental health prevention	Little p policy
Owens (2022) [59]	Case 1	USA (rural county)	Substance use and incarceration	Jail-based reentry for opioid use disorder	Little p policy
FreeBairn (2017) [14]	Case 1	Australia	General health	Health policy decision-making	Big p policy
Lazo-Porras (2020) [60]	Case 1	Peru	Public health, primary care	Diagnosis and management of chronic diseases in primary healthcare	Little p policy

### Characteristics of included documents (*n* = 22 documents)

Documents included codesign studies across multiple continents, Australia/New Zealand (32%), UK/Europe (32%), South America (14%), Africa (9%), and USA/Canada (23%). The types of documents were classified as case studies (77%) which included qualitative description of phases and participant responses, descriptive studies (9%) which included quantitative process evaluation methods, syntheses (4%) which included comparisons of outcomes among different codesign efforts, and opinions (9%) which described codesign activities within a debate or opinion article.

### Health focus and policy type (*n* = 32)

#### Health areas

The focus of health policy ranged widely. Eleven studies (34%) focused on health or social services delivery (e.g., early childhood education, mental health, substance use, tuberculosis care), followed by housing policy (9%), economic development/employment (9%), water supply (6%), built environment (6%), public health (6%), and general health policy (13%).

#### Policy type

Little p policy change was predominant (47%), which included organizational agreements and clinical guidelines. Big p policy (25%) included laws, regulations, or financing changes. Service innovation (25%) included the development of new programs.

### Frameworks and definitions

#### Range of definitions

The most commonly used term to describe efforts was codesign (44%), followed by coproduction (16%), human-centered design (9%), participatory policymaking (6%), design-led policy (6%), participatory communication (3%), public participation (3%), community-engaged evidence synthesis (3%), cocreation (3%), coprioritization (3%), and knowledge-system action analysis (3%) (Table 2).

**Table 2:** Codesign descriptions and characteristics

		Sectors engaged	Steps	User ownership
	*n* = 33	*m* = 4, sd = 1.33	*m* = 5.1, sd = 0.84	*m* = 2.1, sd = 1.94
Codesign	13	4.3	5.8	2.1
Coproduction	5	3.0	4.8	2.4
Human centered design	3	5.0	7.0*	2.0
Participatory policymaking/budgeting	4	3.5	4.3	2.7
Public participation	1	4.0	6.0	3.0
Cocreation, coprioritization	3	4.0	2.7*	2.0
Community engaged	1	5.0	4.0	2.0
Design led policy	2	3.5	5.0	1.0
KASA framework^a^	1	3.0	3.0*	0.0

^*^p < . 05 difference from the mean

^a^Knowledge Action Systems Analysis

### Theories

Referenced theories included formal design-thinking frameworks (44%), political science frameworks (38%), or health services research frameworks (16%). Coders drew from the journal discipline, the department, or the organization of the authors to assign studies to one of these three framework types. Studies referencing design-thinking emphasized the advantages of codesign for developing innovative solutions to intractable health policy problems. A foundational citation found in a reviewed paper included, for example, Verganti [61], *Design-driven innovation, changing the rules of competition by radically changing what things mean.* Political science theories emphasized citizen/resident engagement in public governance. A foundational article cited by reviewed studies included, for example, Mitlin [62], *With and beyond the state—coproduction as a route to political influence, power and transformation for grassroots organizations.* Health services research studies emphasized the use of evidence synthesis or research evidence as a part of social and policy innovation. For example, Richardson [32] describes a health services research study that pivoted to codesign after a researcher-led approach failed to capture sufficient interest and participation.

#### Engagement level of intended beneficiaries (policy users)

The most common level of policy user participation was representative (50%), in which policy users sat on advisory teams but did not have full decision-making power. This was followed by full ownership (25%) in which policy users had voting or comparable influence on decision-making and informant (25%) in which policy users were consulted by were not involved in decision-making. Two studies were deemed to include no involvement (6%) in which policy users were not part of the policy codesign process.

#### Multi-sector involvement

Consumer/community was the most commonly involved sector (84%), followed by government (78%), nonprofits (38%), research and universities (44%), service provider/industry (44%), and philanthropy (6%). The number of sectors within each study ranged from 2 to 7 with a mean of 4 sectors per study.

### Codesign structure

#### Number of phases

The number of phases across studies ranged from 2 to 10 with an average of 5 phases.

**Fig. 2:**
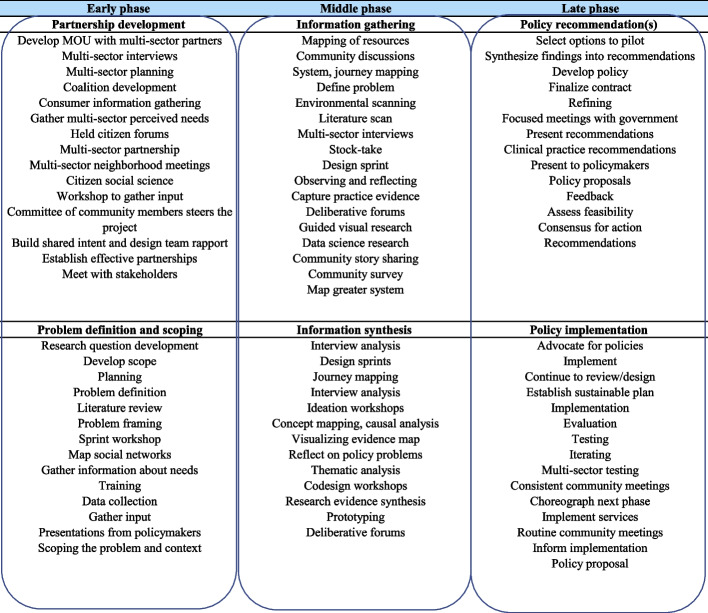
Strategies within phases of policy codesign

Description of phases. Activities within the first phase generally fell into either information gathering (e.g., mapping, literature review) or sector engagement (e.g., coalition-building, developing Memorandums of Understanding). Middle phases tended to include synthesis, presentation, and/or feedback activities (e.g., forums, workshops). Final phases tended to describe a product or final presentation and some studies reflected an intent to engage in continuous policy implementation. We identified a common pattern of linearity in phases: (1) scope the project and build a team, (2) convene stakeholders, (3) gather information from diverse sources, (4) integrate information and prototype solutions, and (5) test for acceptability. Within this general pattern, studies varied significantly in the strategies used to plan, convene, gather information, integrate information, prototype, and test (Fig. 2).

### Perceived benefits

Open coding analysis of the reported benefits of policy codesign across studies yielded five themes: increased knowledge of community needs, community mobilization and equity, feasible policy options, multi-sector alignment, novel ideas, and critical thinking (Fig. 1). To provide context for the authors’ reports of perceived benefits, we provide a study example for each type (Table 3).

**Table 3:** Beneficial outcomes and challenges of policy codesign

	Positive reported outcomes					Reported challenges
Article/case study	Knowledge of community needs	Community mobilization (equity)	Feasible policy options	Multi-sector alignment	Novel ideas, critical thinking	
Banana (2015) [41]	x	x	x	x		Finding community and system individuals willing to commit social capital to being a codesigner and champion, dynamic relationships among community and government
Evans (2016) Case study 1 [19]	x	x	x		x	Emotionally taxing for citizen participants
Hagen (2018) [63] Case study 1	x	x	x	x	x	Finding community members willing to commit social capital and participate in codesign framework, need for flexibility, resource intensive, skepticism and discomfort with ambiguity
Hagen (2018) [63] Case study 2	x				x	Slowness, skepticism from those used to traditional approaches to policy consultation
Hagen (2018) [63] Case study 3			x	x	x	Discomfort with ambiguity, cost and time needed for codesign, skepticism among those used to more traditional policy consultation
Hagen (2018) [63] Case study 4	x	x				Energy and resource intensive, skepticism among those used to traditional policy consultation,
Hagen (2018) [63] Case study 5			x	x		Energy and resource intensive, slowness and flexibility, prototyping complex situations is difficult
Hagen (2018) [63] Case study 6			x		x	Energy (not necessarily resource) intensive, need for flexibility, consistent need for engagement
Hagen (2018) [63] Case study 7	x	x			x	Energy and resource intensive, periods of slowness, consistent need for engagement, skepticism among those used to traditional policy consultation
Holmes (2011) [43] Case study 1	x	x	x		x	Energy and resource intensive, requires strong project management
Holmes (2012) Case study 5	x	x				Energy and resource intensive, consistent need for engagement
Llano-Arias (2015) [45] Case study 1		x				Discomfort among politicians with increased community mobilization, policy implementation difficult
Llano-Arias (2015) [45] Case study 2		x		x		_
Mullins (2021) [46]	x	x				Difficulty recruiting sufficient citizens, need for continuous engagement, consistently negotiating power differences
Munoz-Erickson (2014) [47]						_
van der Bijl-Brouwer (2016) [40]				x	x	Managing multiple stakeholder groups
Bovaird (2012) Case study 2 [48]	x	x				_
Bovaird (2012) Case study 3 [48]	x				x	_
Bovaird (2007) [49] Case study 2	x	x			x	Negotiating between professional and citizen roles
Ostrom (1996) [50]		x	x		x	Supporting citizens to commit social capital to the project, good teamwork within public agencies, regular communication, time and resource intensive
Marchal (2021) [51]	x				x	Those lower in political hierarchies may not feel empowered to speak up without the process intentionally facilitating a sense of safety
Springs (2019) [52]			x		x	-
Spaa (2022) [53]				x	x	-
Richardson (2021) [54]	x	x				The time needed to meaningfully engage residents
Young (2018) [55]	x					-
Bittle (2022) [56]	x	x				Time needed to build and maintain strong relationships. Ensuring participatory approaches engages residents outside of “the usual suspects” who tend to be highly engaged
Lloyd-Williams (2021) [57]	x		x			This effort did not involve consumers/intended beneficiaries and the authors note that equitable solutions would benenfit from more consumer involvement
Goodyear (2022) [58]	x			x	x	Even with codesign, implementation of new approaches will be challenging
Owens (2022) [59]	x	x	x			The array of ideas considered by a codesign process may be limited by the self-selection of participants, i.e., those who are willing to join may already have biases
FreeBairn (2017) [14]			x	x		
Lazo-Porras (2020) [60]			x	x		The time and effort needed goes beyond traditionally accepted timeframes for policy development. Methods must balance scientific knowledge and community needs

An increased knowledge of community needs was noted in 20 studies (63%). A case study reported in Hagen [63] to increase engagement in early childhood education services noted that “the work emphasized how front-line staff and citizens were willing to share their experiences and actively participate in the development and implementation of new ideas” (pg. 42). The codesign process began with in-depth interviews of early childhood education staff and parents and synthesis of subject matter expert knowledge collected through an invited presentation. The synthesized information was brought into an “ideation workshop,” and prototyping solutions were conducted with early childhood education staff and parents.

Community mobilization and equity were a reported benefit in 16 studies (50%). Ostrom [50] noted “In Brazil, many urban neighborhoods that had never undertaken collective action were empowered by the action of government officials to make real decisions and coproduce an urban service that was highly valued” (pg. 1078). The process began with compiling key information on housing and holding meetings to discuss this information in individual neighborhood blocks. The meetings provided a dual function in facilitating discussion between neighbors on key issues related to design and providing information to government city planners about citizen concerns. Subsequent communications and negotiations were informal and ended in a signed agreement from residents about the housing development plan.

The development of more feasible policy options was reported by 13 studies (41%). Lloyd-Williams [57] reported a case study to develop prevention strategies for non-communicable diseases (NCDs) in the UK. They noted that “strategies to prevent premature NCDs therefore potentially represent ‘wicked’ problems…co-production could well be valuable, potentially providing context, relevance, and reality checks regarding feasible strategies” (pg. 19). The presented case study described a process engaging health policy and decision-makers across the UK. Policymakers were engaged in four workshops that iteratively prioritized and narrowed the range of feasible and effective policy options to prevent NCDs.

Improved multisector alignment was noted by 10 studies (31%). Freebairn et al. [14] presented three case examples of participatory dynamic simulation modeling as a strategy to engage “policymakers, researchers, scientists, clinicians, and consumers.” They noted that “an important element of coproduction in these case studies was equal partnering with key stakeholders to negotiate the priority issue” (pg. 10). The approach taken in the case studies involved assembling an initial codesign team to produce a starting model to conceptualize the issue followed by workshops with larger teams to refine conceptual models (i.e., services and casual pathways leading to service outcomes) for the health area.

The introduction of novel ideas and critical thinking was noted in 15 studies (47%). Van der Bijl-Brouwer [40] presented a case study of developing a new service approach to providing mental health crisis response. The author noted the “the method…provides a ‘backbone’ to the human-centered innovation process, by indicating how insights gained through a specific method, e.g., stakeholder interviews, feed into the framing process, and through that that the innovation process” (pg. 13). The approach used was conceptualized as human-centered design, using Dorst’s frame creation methodology [64].

### Relationships between policy user participation and reported benefits

**Fig. 3:**
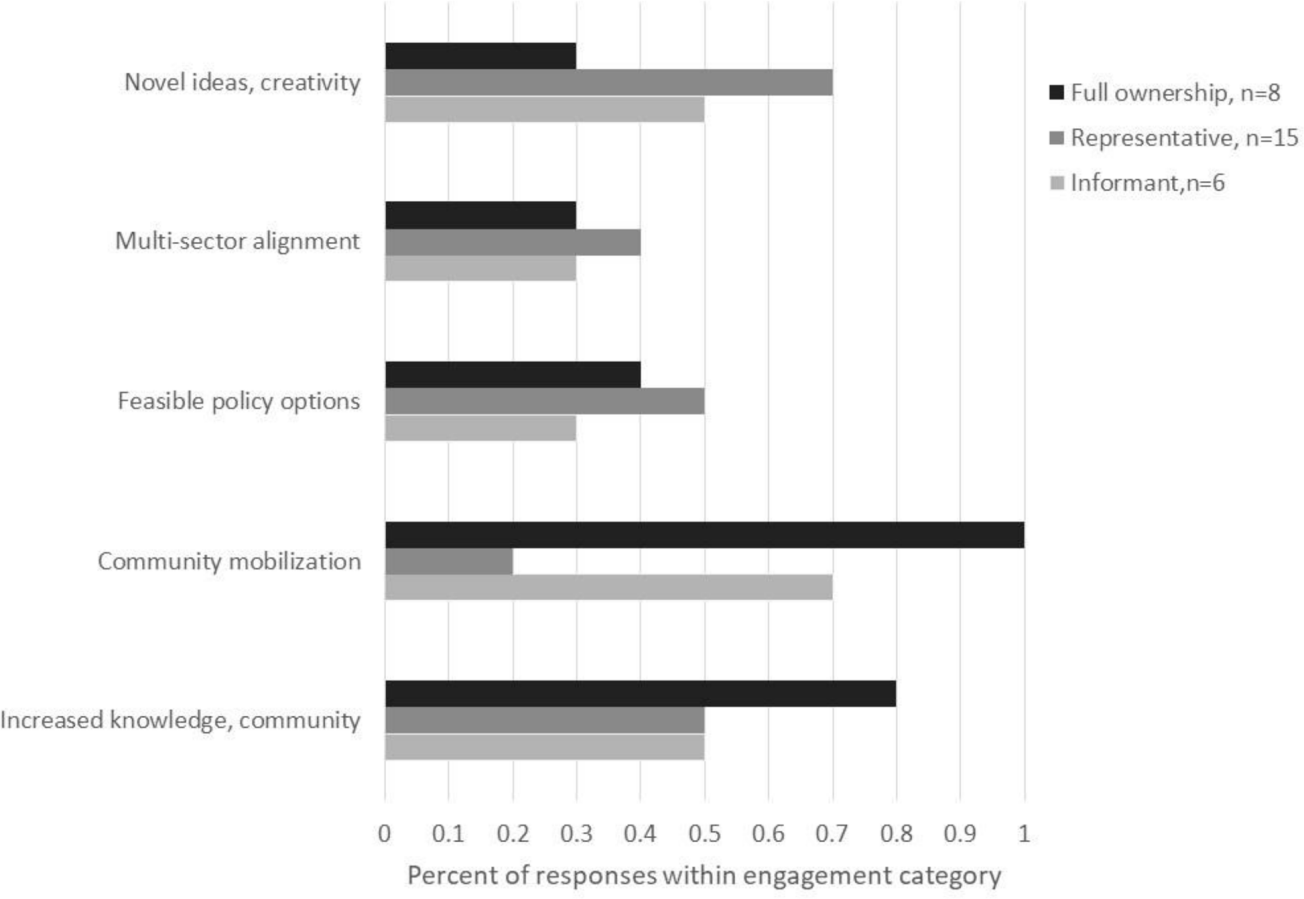
User ownership and policy codesign outcomes

We conducted a descriptive analysis to explore potential associations between the reported benefits of codesign and level of user involvement. Omitting the two cases with no user involvement, we ran a cross tabulation of user involvement level (informant, representative, full ownership) and the five reported outcome areas that emerged from content coding (Fig. 1). We calculated within group percentages of user involvement by outcomes via counting cases within user involvement level and outcome type and dividing by the total cases for that user involvement level. For example, to calculate the percentage of cases at the users as informant level reporting “increased knowledge of community needs,” we summed the cases coded at the informant level reporting this outcome (*n* = 3) and divided it by total number of cases at the informant level (*n* = 6), resulting in 50% of the informant level cases demonstrating “increased knowledge of community needs” (Fig. 3).

Community mobilization and knowledge of community needs were more frequently noted by studies aiming to engage user ownership in policy development. Community mobilization as a beneficial outcome was noted by 100% of the 8 case studies coded at full ownership, compared to 20% of cases coded as only representative involvement (*n* = 8) and 70% of cases coded as informant level involvement (*n* = 6). Novel ideas/critical thinking was most frequently mentioned by cases with users involved at the representative level (70%), compared to full ownership involvement (30%) and informant involvement (50%). No clear difference emerged for multisector alignment or feasible policy options among user involvement level, with both outcomes noted by 50% or less of the assessed case studies.

### Perceived challenges

Studies across disciplinary types and methods repeatedly noted that a codesign approach takes significantly more time and person resources than “typical” policy development approaches (e.g., expert-led consulting and forecasting) [43, 56, 60, 63, 65]. Authors reported the need to allow time for building relationships and trust, particularly when the initiative aims to engage citizens and consumers and where there is a power imbalance among engaged stakeholders. The studies also highlighted the need for codesign teams to have adequate skills in facilitation and project management in proportion to the degree to which efforts aimed to engage multiple sectors, diverse views, and sources of information [40, 43, 60, 63]. Lack of adequate experience or skills in these areas could lead to a loss of perceived credibility, shared sense of mission, and engagement.

Several studies also noted the emotional burden codesign can place upon consumer and citizen participants, particularly when consumers are asked to reflect on poor service delivery or difficult health experiences (e.g., navigating crisis services) [19, 46]. Similarly, authors noted that codesign can be taxing on facilitators who are tasked with maintaining the engagement of diverse participants [46, 50, 63].

Skepticism towards codesign from policymakers and decision-makers and anxiety from stakeholders about the ambiguity of project aims in the early phases of the process were repeatedly noted by authors [41, 45, 63]. Authors also noted the importance of continuous reorientation for engaged stakeholders to the phase and goals of the project and the through-line of synthesized information in final recommendations and policy products [43, 46, 50, 63]. Relatedly, authors noted that the skills needed to facilitate codesign are not routinely found in public administration, posing a challenge for the successful implementation of codesigned policies as well as the broader use of these approaches in routine policy development [49].

## Discussion

We conducted this review to examine how policy codesign is being defined and operationalized in health policy scholarship. We identified a small, growing literature that is expansive in geographical reach and topic area. Health policy codesign is being implemented across continents with somewhat higher representation in the literature among Anglophone countries. The approach is being used to address diverse health topics, including social determinants (housing, economic development), public health, and health services. The maturity of the science across disciplines is at an early stage. Reported outcomes were qualitative and not consistently defined but pointed towards common areas of interest for measuring outcomes.

Efforts to enact little p policy (organizational adoption of practices) was slightly more common than big p policy or program development efforts. Studies were twice as likely to engage policy users as representatives in decision-making than as full owners or as informants. Full ownership approaches tended to be complex, requiring significant resources devoted to community mobilization [41, 50], dialogue, and voting [56]. Representative approaches tended to be more time-limited, using discrete engagement events such as workshops, to engage participants from multiple sectors [19, 43, 63]. Informant approaches were focused on improving the robustness of information available to policymakers in their decision-making.

Relying on the author-reported benefits of codesign, we identified a trend association between the use of full ownership approaches and the reported level of community mobilization and knowledge of community needs. We also identified higher reported benefits in novel ideas within representative ownership approaches. Because the reported outcomes were purely descriptive, we identify this as a productive area for future research. Potential trade-offs between community mobilization and novel ideas suggest different policy codesign structures may yield different benefits and should be used to solve different types of policy problems. This aligns with the general guidance of research and policy participation frameworks [66]. In Gupta’s split ladder of participation framework, for example, the model suggests only using complex, policy user intensive efforts when the health topic is controversial, with little agreement among sectors in values or beliefs about the relevant research evidence.

We did not attempt to examine associations between the linearity or phases of policy codesign efforts and outcomes because of the variation in approaches. We observed general trends; however, all approaches identified a phase in the process in which information had to be “put together” in some way to propose a policy solution, with these solutions coming middle to late in the policy formation process. Accordingly, a common feature noted across the articles reviewed and that spanned disciplinary approaches, was the greater amount of time required to facilitate a policy codesign effort when compared to traditional timelines. Studying the trade-offs between timeframe and the development of trusting relationships is an important area of future research. It is likely that long timeframes could hurt the acceptability or scalability of policy codesign and researchers will be motivated to find ways to shorten timeframes to improve efficiencies. Findings from the articles reviewed here suggests facilitation expertise might be a factor in reducing timeframes; on the other hand, studies cautioned that imposing urgency when forming partnerships with individuals marginalized by race, poverty, and/or disability will further harm those individuals. The intended benefits of a proposed policy should not outweigh the potential for harm among individual participants in the codesign process [67, 68]. Distilling insights from experienced community/multi-sector facilitators and examining time-limited activities and methods from participatory design could yield a set of guiding principles for making these tradeoff decisions. Researchers studying policy codesign can assist by clearly documenting activities within phases, time devoted to phases, and participant perceptions of the codesign process using participatory process measures [69–71].

Knowledge gathering and integration, possibly the area of greatest interest for implementation science, was infrequently described in meaningful detail among the reviewed studies. Traditionally, the field of participatory design and codesign has not considered the use of research evidence as a core element of knowledge synthesis. Although as formally trained designers are moving more into health services research, the use of research evidence within design engagements is becoming more prevalent [72]. In our review, research evidence was formally presented in the policy codesign efforts of facilitator teams that came from health services research organizations [45, 59, 65], whereas research use was less formally introduced or not introduced by facilitator teams with participatory design or public policy backgrounds. The use of research evidence within anti-racist and decolonizing movements within health services research is currently contentious; a key question for the field is how to make appropriate use of research evidence without imposing this use on communities [73, 74]. Policy codesign, and codesign broadly, provides a promising framework and set of methods for resolving this tension.

In codesign, participants can request information to round out their view of a topic, including the research evidence, without this needing to be pre-selected or imposed by a facilitator team. In Owens [59], for example, the facilitator team used an established systems-design model, Theory U, which does not require a formal “research evidence” component. In the project, the codesign participants requested a review of best practices in jail-based reentry for opioids and a research team conducted a rapid evidence review (RER) on the topic. The RER was translated into a 10-min video and research brief and sent back to the team to review and discuss. Interestingly, the paper notes that when asked if they “learned something new,” no one on the team noted the RER findings. However, the cross-service model developed strongly resembled existing evidence-informed recommendations while also including innovative components (e.g., peer navigation support). This and other literature suggest research evidence can be a valuable information source but should not dominate and sometimes may not be necessary (or could be harmful) when designing policies and systems to solve intractable health problems [75, 76]. The promise of policy codesign, albeit in an infant stage, is having a framework for selecting and synthesizing information and engaging partners to create the most transformative or effective policy solution possible for that moment.

### Limitations

Because we limited our review to policy efforts, we did not review a much larger literature reviewing the use of codesign in health services research and in health service program development. Review of these efforts already exists [26, 77, 78]. We also excluded citizen-led mobilization and advocacy efforts even though these actions form an important part of the health services improvement landscape.

## Conclusions

Policy codesign is an emerging and converging area of study that shows promise for reconciling some of the competing demands on governments to engage citizens, be responsive to community needs, and steward the effective use of public funds to improve health. Studies of policy codesign show little consistency in specific methods but are coalescing around phases in which actual policy development comes after a meaningful number of activities devoted to coalition building, information gathering, and synthesis. Outcomes reported by study authors suggest key areas of measurement for continuing to study and advance policy codesign including community mobilization and the development of novel ideas.

## Data Availability

Coding sheets and Search Protocol are available at https://osf.io/sw2fr/.

## Abbreviations

EB: Evidence-based
EBI: Evidence-based interventions
NPG: New Public Governance
PRISMA-ScR: Preferred Reporting Items for Systematic reviews and Meta-Analyses extension for Scoping Reviews
NCDs: Non-communicable diseases
RER: Rapid evidence review
